# Insecticidal toxicities of carvacrol and thymol derived from *Thymus vulgaris* Lin. against *Pochazia shantungensis* Chou & Lu., newly recorded pest

**DOI:** 10.1038/srep40902

**Published:** 2017-01-20

**Authors:** Jun-Hwan Park, Ye-Jin Jeon, Chi-Hoon Lee, Namhyun Chung, Hoi-Seon Lee

**Affiliations:** 1 Department of Bioenvironmental Chemistry, Chonbuk National University, Jeonju 54896, Korea; 2 Division of Research Planning, National Research Institute of Health, Centers for Disease Control & Prevention, Chungbuk 28159, Korea; 3 Department of Biosystems and Biotechnology, College of Life Sciences and Biotechnology, Korea University, Seoul 02841, Korea

## Abstract

The insecticidal toxicities of five essential oils against *Pochazia shantungensis* adults and nymphs, newly recorded pests, were evaluated. The LC_50_ values of *Thymus vulgaris, Ruta graveolens, Citrus aurantium, Leptospermum petersonii* and *Achillea millefolium* oils were recorded as 57.48, 84.44, 92.58, 113.26 and 125.78 mg/L, respectively, against *P. shantungensis* nymphs using the leaf dipping bioassay, and 75.80, 109.86, 113.26, 145.06 and 153.74 mg/L, respectively, against *P. shantungensis* adults using the spray bioassay method. Regarding volatile components identified in *T. vulgaris* oil, the LC_50_ values of carvacrol and thymol using the leaf dipping bioassay against *P. shantungensis* nymphs were 56.74 and 28.52 mg/L, respectively. The insecticidal action of *T. vulgaris* oil against *P. shantungensis* could be attributed to carvacrol and thymol. Based on the structure-toxicity relationship between thymol analogs and insecticidal toxicities against *P. shantungensis* nymphs similar to the LC_50_ values against *P. shantungensis* adults, the LC_50_ values of thymol, carvacrol, citral, 2-isopropylphenol, 3-isopropylphenol, and 4-isopropylphenol were 28.52, 56.74 and 89.12, 71.41, 82.49, and 111.28 mg/L, respectively. These results indicate that the insecticidal mode of action of thymol analogs may be largely attributed to the methyl functional group. Thymol analogues have promising potential as first-choice insecticides against *P. shantungensis* adults and nymphs.

*Pochazia shantungensis* Chou & Lu. is a hemipterous insect that belongs to the Ricaniidae insect family, which includes about 400 described species in over 40 genera^1^. *P. shantungensis* is a newly recorded pest that is economically devastating for various trees including apple, blueberry, peach and persimmon, mainly in Wanjugun, Korea^2^. *P. shantungensis* was first recorded in Chungcheongnamdo in 2010^3^. In later outbreaks of exotic invasive insects, *P. shantungensis* has subsequently occurred sporadically in several other provinces across Korea^2^,^3^. In spite of this, the species identification has not yet been confirmed, mostly due to the impossibility of identifying the genital characteristics of externally similar species^2^. As such, an insecticide has yet to be developed for long-term control of *P. shantungensis* adults and nymphs, despite the urgent demand^4^. Discovering selective and natural insecticides that are safe for the environment and other organisms is essential for the management of *P. shantungensis*.

Plant oil is a very complex mixture that can contain approximately 30–65 constituents at various concentrations^3^,^5^,^6^. Two or three major constituents are featured at 25–60% of the concentration compared to other components present in trace quantities^3^. Acetovanillone (47.98%) and 2′-hydroxy-5′-methoxyacetophenone (49.23%) are the major components of *Cynanchum paniculatum* oil^7^, menthol (59%) and menthone (19%) are the major components of *Mentha piperita* oil^8^, and carvone (59.79%) and limonene (25.40%) are the major components of *Mentha spicata* oil^9^. The essential oils and major components derived from plants are significant insecticidal activity against diverse insect species and have been developed as ecologically potential pesticides^3^,^7^,^8^,^9^,^10^.

So for no report has been received about the insecticidal toxicities of *Thymus vulgaris* oil-derived constituents against *P. shantungensis*. Therefore, the aims of the present study were first to investigate the insecticidal properties of *T. vulgaris* oil-derived components against *P. shantungensis* adults and nymphs, and then to determine the structure-activity relationship between thymol analogs and insecticidal toxicities.

## Results and Discussion

This study was undertaken within the framework of a more general study involving the natural products for insecticidal toxicities against *P. shantungensis* adults and nymphs. Essential oils of *Achillea millefolium* flowers, *Citrus aurantium* fruits, *Leptospermum petersonii* leaves, *Ruta graveolens* leaves and *T. vulgaris* leaves were analyzed (Table 1). The yields of *A. millefolium, C. aurantium, L. petersonii, R. graveolens* and *T. vulgaris* oils were 0.658, 1.451, 0.984, 0.924, and 1.122%, respectively. The insecticidal toxicities of the five oils against *P. shantungensis* adults and nymphs were evaluated after 48 and 72 h exposure (Table 2). From the leaf dipping and spray bioassays against *P. shantungensis* adults and nymphs, the insecticidal responses and the LC_50_ values increased from 48 to 72 h exposure. The LC_50_ values of *T. vulgaris, R. graveolens, C. aurantium, L. petersonii* and *A. millefolium* oils at 72 h exposure were 75.80, 109.86, 113.26, 145.06 and 153.74 mg/L, respectively, in the spray bioassay against *P. shantungensis* adults, and 57.48, 84.44, 92.58, 113.26 and 125.78 mg/L, respectively, in the leaf dipping bioassay against *P. shantungensis* nymphs. Based on the LC_50_ values against *P. shantungensis* adults and nymphs, *T. vulgaris* oil had the highest insecticidal toxicity followed by *R. graveolens, C. aurantium, L. petersonii* and *A. millefolium* oils. The insecticidal toxicity of *T. vulgaris* oil against *P. shantungensis* nymphs was about 1.3-fold more than that against *P. shantungensis* adults. There was no insect mortality in the distilled water treatment (negative control) of *P. shantungensis* adults and nymphs. Differences in the insecticidal toxicities of plant-derived oils may be explained on the basis of species-specific responses to plant species, phytochemicals, and the weight and size of *P. shantungensis* adults and nymphs^11^.

To further explore the insecticidal toxicities of the five essential oils against *P. shantungensis* adults and nymphs, the components of *A. millefolium, C. aurantium, L. petersonii, R. graveolens*, and *T. vulgaris* oils were investigated by GC-MS analysis. The identified components, together with the percentages present in the essential oils are displayed in Table 3. The major components were α-pinene (15.49%), β-caryophyllene (13.35%), sabinene (11.12%), camphor (9.65%), 1,8-cieole (9.37%), bornyl acetate (5.88%), and 2,2-dicyclohexylmalononitrile (5.58%) in *A. millefolium* oil; limonene (87.75%), citral (3.21%), limonene oxide (2.29%), and (−)-carveol (1.75%) in *C. aurantium* oil; citral (48.12%), β-citronellal (19.50%), isopulegol (8.14%), geraniol (4.64%), and linalool (3.33%) in *L. petersonii* oil, and thymol (23.34%), undecyl trichloroacetate (18.52%), methyltridecyl pentanoate (16.18%), and 2,2-dimethylpropanoic acid (7.03%), 2-acetoxytetradecane (6.98%), palmitic acid (5.07%), and methyl linolenate (4.78%) in *R. graveolens* oil. The major components in *T. vulgaris* oil were thymol (40.04%), ρ-cymene (29.97%), γ-terpinene (8.17%), linalool (4.99%), terpinolene (3.26%), α-pinene (2.84%), β-caryophyllene (2.50%), carvacrol (2.45%), limonene (1.25%), α-phellandrene (1.20%), myrcene (0.91%), camphene (0.89%), and caryophyllene oxide (0.21%). Together, thymol, ρ-cymene and γ-terpinene made up 78.18% of *T. vulgaris* oil. The volatile components consisted of 8 monoterpene hydrocarbons (camphene, α-pinene, limonene, myrcene, terpinolene, γ-terpinene, α–phellandrene, and ρ-cymene), 1 monoterpene alcohol (linalool), 2 monoterpene phenols (carvacrol and thymol) and 2 sesquiterpene hydrocarbons (β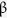-caryophyllene and caryophyllene oxide). Venskutonis^9^ reported that the main chemicals identified in the *T. vulgaris* oil were borneol (0.98%), camphene (0.60%), carvacrol (2.81%), β-caryophyllene (2.39%), 1,8-cineol (0.96%), *p*-cymene (25.2%), myrcene (1.93%), linalool (2.86%), α-pinene (1.16%), 1-octen-3-ol (1.19%), α-terpinene (1.02%), γ-terpinene (6.37%), α-thujene (1.50%) and thymol (42.27%). The fact that the essential oil of *T. vulgaris* leaves dried more at 45 °C than at 30 °C can be explained mainly by the increase of thymol by 8% and carvacrol by 12%, while the quantity of γ-terpinene was decreased by 4.9%^9^. In previous and present studies, the quantities of volatile chemicals derived from *T. vulgaris* were affected by the environmental conditions, including harvest time, genotype, storage period, handling method and intraspecific variability, and the experimental conditions, which included the extraction method, extracted plant parts, and plant tissue drying temperature^12^,^13^.

**Figure 1:**
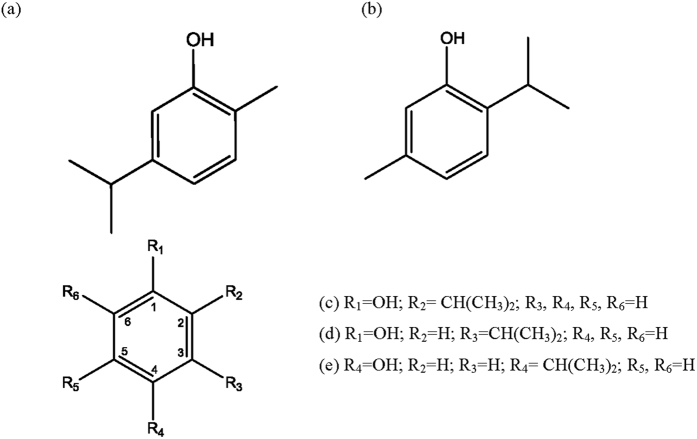
Structures of thymol structural related analogs. (**a**) 2-isopropyl-5-methylphenol (thymol); (**b**) 5-isopropyl-2-methylphenol (carvacrol); (**c**) 2-isopropylphenol; (**d**) 3-isopropylphenol; (**e**) 4-isopropylphenol.

The insecticidal toxicities of 34 major commercial components (bornyl acetate, camphene, camphor, carvacrol, (−)-carveol, (+)-carvone, β-caryophyllene, caryophyllene oxide, β-citronellal, citral, 1,8-cineole, *p*-cymene, decyl chloroformate, dodecanoic acid, β-farnesene, geranyl acetate, geraniol, isopulegol, linalool, limonene, limonene oxide, methyl linolenate, myrcene, myristic acid, palmitic acid, α-phellandrene, α-pinene, pivalic acid, sabinene, α-terpineol, γ-terpinene, 4-terpineol, terpinolene, and thymol) derived from the five essential oils were evaluated using spray and leaf dipping bioassays against *P. shantungensis* adults and nymphs (Table 4). Based on the LD_50_ values against *P. shantungensis* nymphs, the LC_50_ values of thymol, carvacrol and citral identified in *C. aurantium, L. petersonii, R. graveolens*, and *T. vulgaris* oils using the leaf dipping bioassay were 28.52, 56.74 and 89.12 mg/L respectively. Using the spray bioassay against *P. shantungensis* adults, the LC_50_ values of thymol, carvacrol and citral were 42.12, 75.62, and 102.74 mg/L, respectively. The insecticidal toxicity of thymol against *P. shantungensis* nymphs and adults was approximately 1.8–3.3 times greater than that of carvacrol and citral. In contrast, the other components (β-caryophyllene, camphene, caryophyllene oxide, ρ-cymene, linalool, limonene, myrcene, α-phellandrene, α-pinene, terpinolene, γ-terpinene, sabinene, β-pinene, camphor, (−)-carveol, geraniol, and bornyl acetate) did not exhibit any insecticidal toxicity against *P. shantungensis* adults and nymphs (data not shown). The insecticidal toxicities of the essential oils appear to be connected to their chemical composition. The insecticidal toxicities of *T. vulgaris* and *R. graveolens* oils could be due to the existence of thymol and carvacrol, which exhibited the greatest insecticidal toxicities. The essential oils of *C. aurantium* and *L. petersonii* contain citral, which showed insecticidal toxicities against *P. shantungensis* adults and nymphs, however its toxicity was weaker than thymol and carvacrol. Furthermore, *P. shantungensis* nymphs were more susceptible to *T. vulgaris* oil, carvacrol and thymol, when compared to *P. shantungensis* adults (Fig. 1). In a previous study, the differential susceptibility shown by *P. shantungensis* adults and nymphs to thymol and carvacrol was attributed to differences in the weights and sizes of *P. shantungensis* adults and nymphs, as well as the potential to detoxify glutathione *S*-transferase and hydrolase^7^,^8^,^9^. The synergetic effect of thymol combined with carvacrol has previously been reported for other insects, such as beetles^14^ and lepidopterans^6^. Medeiros *et al*.^15^ suggested that thymol and carvacrol to different species of insects are connected with the insecticidal effect of these monoterpenes on the cells of target insects, since they cause disorganization in the cell membrane, leading it to lose permeability^15^. In contrast, although the *A. millefolium* oil did not contain thymol, carvacrol and citral, the insecticidal properties of *A. millefolium* against *P. shantungensis* adults and nymphs could be due to internal synergy or blend effect of their constituents. Previous study reported internal synergy or a blend effect of the main constituents of plant oil for *Ocimum kenyenst*^16^, *Zanthoxylum armatum*^17^ and *Plectranthus marruboides*^18^ oils against the mosquito species, *Aedes aegypti* and *Anopheles gambiae*. Our results indicate that some terpenes containing the other tested components may correlate with the detoxification mechanisms of *P. shantungensis* adults and nymphs by several terpenes. Treatment with terpenes of *Melia azedarach* against *Spodoptera littoralis* can significantly increase the activities of α-esterase and β-esterase, which are important detoxifying enzymes^19^ and significant decreased the acid phosphatases, alkaline phosphatases, adenosine triphosphatases and the lactate dehydrogenase of *Cnaphalocrocis medinalis*^20^.

In order to establish the structure-toxicity relationship between thymol analogs and insecticidal toxicities against *P. shantungensis* adults and nymphs, thymol, carvacrol, 2-isopropylphenol, 3-isopropylphenol, and 4-isopropylphenol were selected as thymol analogs for testing (Fig. 1). The insecticidal toxicities of thymol structurally related analogs and how activity varies with structure were investigated using leaf dipping and spray bioassays against *P. shantungensis* adults and nymphs (Table 4). Based on the LC_50_ values of thymol analogs against *P. shantungensis* nymphs, the LC_50_ values of thymol, carvacrol, 2-isopropylphenol, 3-isopropylphenol, and 4-isopropylphenol using the leaf dipping bioassay were 28.52, 56.74, 71.41, 82.49, and 111.28 mg/L, respectively. Using the spray bioassay against *P. shantungensis* adults, the LC_50_ values of thymol, carvacrol, 2-isopropylphenol, 3-isopropylphenol, and 4-isopropylphenol were 42.12, 75.62, 85.77, 104.65, and 122.36 mg/L, respectively. The insecticidal toxicity of thymol against *P. shantungensis* nymphs and adults was approximately 1.80–3.90 times greater than that of carvacrol, citral, 2-isopropylphenol, 3-isopropylphenol, and 4-isopropylphenol. While the functional group in thymol was necessary for insecticidal toxicity, the removal of the methyl functional group reduced in insecticidal toxicity. Furthermore, the position of the methyl and isopropyl functional group in the phenol ring altered insecticidal toxicity. These results indicate that the insecticidal mode of action of thymol analogs may be largely attributable to the methyl functional group. This observation contrasts to an earlier finding that the isopropyl functional group in thymol analogs is key in imparting insecticidal toxicity against stored-food pests^21^.

The present results implicate *T. vulgaris* oil, thymol and thymol structurally related analogs as promising natural products of insecticides against exotic insects. Others have found visual evidence of leaf phytotoxicity caused by *T. vulgaris* oil to the host plant of *P. shantungensis*, grape leaf^22^. The LD_50_ values of carvacrol, thymol, and *T. vulgaris* oil against rat are 810, 980 and 2,840 mg/kg, respectively, by oral administration and the dermal LD_50_ value of thymol and *T. vulgaris* oil exceeds 2,000 mg/kg against rat and 5,000 mg/kg against rabbit, respectively^23^. These results suggest that *T. vulgaris* oil, carvacrol, thymol and thymol analogs have a relatively low acute toxicity in mammals.

Our study is the first to investigate the insecticidal toxicities of *T. vulgaris* oil, thymol and thymol analogs against *P. shantungensis* adults and nymphs. Considering the fact that *T. vulgaris* is a very inexpensive plant to acquire and is easily cultivated, and are not barriers for the commercial development of carvacrol, thymol, and *T. vulgaris* oil isolated from *T. vulgaris*. Further study is required to decrease the human toxicity of the *T. vulgaris* oil, thymol and thymol analogs and establish the insecticidal mode of action of thymol analogs against *P. shantungensis* adults and nymphs.

## Materials and Methods

### Chemicals and material preparation

Bornyl acetate (99%), camphene (95%), camphor (96%), carvarol (98%), (−)-carveol (95%), (+)-carvone (96%), β-caryophyllene (98.5%), caryophyllene oxide (95%), β-citronellal (95%), citral (95%), 1,8-cineole (99%), *p*-cymene (99%), decyl chloroformate (97%), dodecanoic acid (98%), β-farnesene (90%), geranyl acetate (97%), geraniol (98%), isopulegol (98%), linalool (97%), limonene (97%), limonene oxide (97%), methyl linolenate (99%), myrcene (90%), myristic acid (99%), palmitic acid (99%), α-phellandrene (85%), α-pinene (98%), pivalic acid (99%), sabinene (75%), α-terpineol (90%), γ-terpinene (97%), 4-terpineol (95%), terpinolene (90%), and tymol (99%) were obtained from Sigma-Aldrich (St. Louis, MO, USA). *Achillea millefolium* L. flowers, *Citrus aurantium* L. fruits, *Leptospermum petersonii* F. M. Bailey leaves, *Ruta graveolens* L. leaves, and *Thymus vulgaris* L. leaves were collected from a local store in Chonju, Korea. Sample specimens were authenticated by Jeongmoon Kim at Chonbuk National University, Korea. Essential oils of the five plants were obtained by steam distillation extraction, and finally dried over Na_2_SO_4_ to extract the pure essential oils (Table 1).

### Insects and bioassays

*P. shantungensis* adults and nymphs were collected from persimmon trees in Wanjugun, Korea and classified the fourth instar stages of *P. shantungensis* nymphs and adults as detailed elsewhere^2^,^4^. The insecticidal toxicities of the five essential oils against *P. shantungensis* adults and nymphs were assessed (Table 2). Experimental protocols were approved by the Korea National Institute of Agricultural Sciences and the Korea Centers for Disease Control and Prevention. Using the leaf-dipping method, the insecticidal toxicities of *A. millefolium, C. aurantium, L. petersonii, R. graveolens* and *T. vulgaris* oils were evaluated against *P. shantungensis* nymphs. Insecticidal toxicities against *P. shantungensis* nymphs were evaluated according to a prior bioassay method^8^. Eight dilutions of insecticidal constituent (1000 to 50 mg/L) were prepared by dissolving in distilled water. Rose leaf disk of sharon (3 cm) were dipped into each test sample for 4 min and allowed to dry. The treated leaf was placed into a petri dish (60 × 15 mm) and 30 nymphs were released. The nymphs affected by this treatment were evaluated for 48 and 72 h after treatment. All treatments were repeated twice at 21 °C. The insecticidal toxicity of each sample was evaluated against *P. shantungensis* adults with the spray bioassay^8^. Eight concentrations (1000 to 50 mg/L) of the insecticidal constituents were diluted in distilled water. An insect square dish (15 × 15 × 20 cm) which contained 20 adults, was sprayed with 200 μL of treatment solutions to run off with a hand-held sprayer. Assessment was carried out 24 and 48 h after treatment by counting the normal insects. The tests were conducted with two replicates and incubated at 21 °C.

### Gas chromatography-mass spectrometry

The components of the essential oil extracted from *T. vulgaris* leaves were quantified using the Hewlett-Packard HP 6890 and H5973IV series (Agilent, Santa Clara, CA, USA) and were separated with HP-Innowax capillary column and DB-5 column (0.25 mm i.d. × 0.25 μm thickness × 2,990 cm L.). The conditions of the column were as follows: Helium at 0.75 mL/min; column temperature (51 to 201 °C) at 2 °C/min; injector temperature (211 °C); split ration (48:1); ion source temperature (231 °C); ionization potential (70e V); and mass spectra range (50–800 amu). The components of *T. vulgaris* oils were evaluated according to retention times, retention indices, and mass spectra and were identified by comparison with a spectrum library (Table 3). The relative composition of each *T. vulgaris* oil constituent (%) was measured by comparison with internal standards.

### Statistical analysis

Data obtained for each dose response bioassay were subjected to probit analysis. The median lethal concentration (LC_50_) value and the slope of the regression lines were calculated using the statistical package SPSS, version 12.0 for Windows.

## Additional Information

**How to cite this article**: Park, J.-H. *et al*. Insecticidal toxicities of carvacrol and thymol derived from *Thymus vulgaris* Lin. against *Pochazia shantungensis* Chou & Lu., newly recorded pest. *Sci. Rep.* **7**, 40902; doi: 10.1038/srep40902 (2017).

**Publisher's note:** Springer Nature remains neutral with regard to jurisdictional claims in published maps and institutional affiliations.

## Figures and Tables

**Table 1:** List of five plants tested and yields of essential oils (Yield (%) = (Dried weight of essential oil/dried weight of sample) × 100).

Plant species	Family name	Tissue sampled	Yield (%)
*Achillea millefolium* L.	Asteraceae	flowers	0.658
*Citrus aurantium* L.	Rutaceae	fruits	1.451
*Leptospermum petersonii* F. M.	Myrtaceae	leaves	0.984
*Ruta graveolens* L.	Rutaceae	leaves	0.924
*Thymus vulgaris* L.	Lamiaceae	leaves	1.122

**Table 2:** Insecticidal toxicities of five essential oils against *P. shantungensis* adults and nymphs at 48 and 72 h exposure times (LC_50_ is the average of 3 determinations, with 30 nymphs and 20 adults per replication).

Plant species	Times (h)	Stage	LC_50_ (95% CI) (mg/L)	LC_90_ (95% CI) (mg/L)	Slope ± SE	χ^2^ (df, *P*)
*Achillea millefolium*	48	Nymphs	175.36 (164.30–192.34)	321.85 (278.58–363.65)	4.24 ± 0.54	13.087 (9, 0.159)
		Adults	186.25 (167.17–216.40)	344.01 (302.71–384.83)	4.77 ± 0.76	2.028 (5, 0.567)
	72	Nymphs	125.78 (107.04–144.47)	295.63 (251.52–348.74)	2.67 ± 0.50	7.658 (7, 0.364)
		Adults	153.74 (130.22–168.54)	310.85 (269.60–353.03)	4.44 ± 0.98	1.643 (6, 0.949)
*Citrus aurantium*	48	Nymphs	118.92 (107.50–130.78)	288.36 (242.84–325.66)	3.69 ± 0.49	9.361 (8, 0.313)
		Adults	156.24 (137.03–176.48)	306.76 (260.59–357.97)	4.11 ± 0.91	1.020 (5, 0.797)
	72	Nymphs	92.58 (84.10–104.01)	205.24 (174.76–243.89)	4.12 ± 0.71	1.971 (6, 0.922)
		Adults	113.26 (97.91–129.13)	280.21 (241.79–315.32)	3.30 ± 0.48	4.315 (5. 0.505)
*Leptospermum petersonii*	48	Nymphs	154.31 (138.86–177.13)	298.25 (257.98–331.83)	4.31 ± 0.68	2.198 (5, 0.821)
		Adults	177.56 (165.10–197.61)	346.16 (273.58–406.17)	4.88 ± 0.75	2.866 (6, 0.825)
	72	Nymphs	113.26 (100.65–125.14)	234.57 (199.72–262.90)	3.34 ± 0.50	3.683 (8, 0.885)
		Adults	145.06 (128.87–167.76)	283.90 (237.47–332.61)	3.64 ± 0.49	2.244 (5, 0.815)
*Ruta graveolens*	48	Nymphs	114.23 (99.90–129.09)	240.42 (208.33–274.18)	2.94 ± 0.47	4.459 (8, 0.814)
		Adults	146.74 (125.80–163.64)	277.85 (238.57–315.48)	4.06 ± 0.74	2.687 (5, 0.611)
	72	Nymphs	84.44 (76.71–93.21)	159.53 (138.09–191.87)	3.72 ± 0.48	4.842 (8, 0.774)
		Adults	109.86 (95.67–122.98)	258.44 (223.68–297.40)	4.01 ± 0.61	1.605 (5, 0.808)
*Thymus vulgaris*	48	Nymphs	78.54 (67.96–89.65)	155.97 (132.75–186.02)	3.23 ± 0.51	4.570 (5, 0.471)
		Adults	108.62 (94.60–127.77)	274.87 (227.14–313.14)	3.81 ± 0.56	3.375 (5, 0.497)
	72	Nymphs	57.48 (50.34–65.74)	122.24 (105.24–153.40)	3.06 ± 0.40	3.320 (7, 0.854)
		Adults	75.80 (61.90–86.23)	139.93 (122.02–166.61)	3.89 ± 0.56	4.095 (5. 0.393)
Negative Control	48	Nymphs	—5		—	—
		Adults	—		—	—
	72	Nymphs	—		—	—
		Adults	—		—	—

**Table 3:** Volatile components of *T. vulgaris* oil identified by GC-MS (RI, retention indices in elution order from the DB-5 column; RT, comparison with pure standard retention time; MS, mass spectrometry).

NO	Constituent	*A. millefolium*	*C. aurantium*	*L. petersonii*	*R. graveolens*	*T. vulgaris*	Identification method
1	α-Pinene	15.49	1.10	0.95	—	2.84	RI, RT, MS
2	Sabinene	11.12	0.21	—	—	—	RI, RT, MS
3	1,8-Cineole	9.37	—	—	—	—	RI, RT, MS
4	2,2-Dicyclohexylmalononitrile	5.58	—	—	—	—	RI, RT, MS
5	Camphene	—	—	—	—	0.89	RI, RT, MS
6	Camphor	9.65	—	—	—	—	RI, RT, MS
7	*p*-Mentha-2,8-dienol	—	0.67	—	—	—	RI, RT, MS
8	Limonene oxide	—	2.29	—	—	—	RI, RT, MS
9	Isopulegol	—	—	8.14	—	—	RI, RT, MS
10	β-Citronellal	—	—	19.50	—	—	RI, RT, MS
11	α-Terpineol	—	0.31	2.07	—	—	RI, RT, MS
12	4-Terpineol	4.81	—	5.17	—	—	RI, RT, MS
13	(−)-Carveol	—	1.75	—	—	—	RI, RT, MS
14	(+)-Carvone	—	—	—	2.46	—	RI, RT, MS
15	Myrcene	—	0.86	0.87		0.91	RI, RT, MS
16	Thymol	—	—	—	23.34	40.04	RI, RT, MS
17	Citral	—	3.21	48.12	—	—	RI, RT, MS
18	Geraniol	—	—	4.64	—	—	RI, RT, MS
19	Bornyl acetate	5.88	—	—	—	—	RI, RT, MS
20	Undecyl trichloroacetate	—	—	—	18.52	—	RI, RT, MS
21	α-Phellandrene	—	—	—	—	1.20	RI, RT, MS
22	Geranyl acetate	2.39	0.65	—	—	—	RI, RT, MS
23	β-Caryophyllene	13.35	—	—	—	2.50	RI, RT, MS
24	2-Acetoxytetradecane	—	—	—	6.98	—	RI, RT, MS
25	β-Farnesene	2.38	—	—	—	—	RI, RT, MS
26	ρ-Cymene	2.07	—	1.30	2.29	29.97	RI, RT, MS
27	Germacrene D	4.63	—	—	—	—	RI, RT, MS
28	Limonene	3.94	87.75	—	—	1.25	RI, RT, MS
29	Decyl chloroformate	—	—	—	3.01	—	RI, RT, MS
30	Viridiflorene	—	—	2.14	—	—	RI, RT, MS
31	Dodecanoic acid	—	—	—	1.25	—	RI, RT, MS
32	γ-Terpinene	—	—	1.29	—	8.17	RI, RT, MS
33	Pivalic acid	—	—	—	7.03	—	RI, RT, MS
34	Methyltridecyl pentanoate	—	—	—	16.18	—	RI, RT, MS
35	Myristic acid	—	—	—	3.48	—	RI, RT, MS
36	Palmitic acid	—	—	—	5.07	—	RI, RT, MS
37	Terpinolene	—	—	—	—	3.26	RI, RT, MS
38	5-(1-Bromo-1-methyl-ethyl)-2-methyl-cyclohexanol	—	—	2.37	—	—	RI, RT, MS
39	Methyl linolenate	—	—	—	4.78	—	RI, RT, MS
40	Linalool	3.75	0.13	3.33	—	4.99	RI, RT, MS
41	Carvacrol	—	—	—	—	2.45	RI, RT, MS
42	Caryophyllene oxide	—	—	—	—	0.21	RI, RT, MS
	Major Grouped Compounds						
	Acid	—	—	—	16.83	—	
	Alkan	—	—	—	6.98	—	
	Ketone	—	—	0.99	—	—	
	Monoterpene alcohol	8.56	2.86	28.69	—	4.99	
	Monoterpene esters	8.27	0.65	—	42.49	—	
	Monoterpene ethers	9.37	—	—	—	—	
	Monoterpene hydrocarbons	32.62	89.92	4.41	4.75	45.49	
	Monoterpene phenol	—	—	—	23.34	45.49	
	Nitrile	5.58	—	—	—	—	
	Oxygenated monoterpens	9.65	5.50	61.29	—	—	
	Sesquiterpene alcohol	—	—	2.37	—	—	
	Sesquiterpene hydrocarbons	20.36	—	2.14	—	2.71	
	**Total**	**94.41**	**98.93**	**99.89**	**94.39**	**98.68**	

**Table 4:** Insecticidal toxicities of major commercial components of five essential oils and thymol analogs against *P. shantungensis* adults and nymphs at 72 h exposure time (LC_50_ is the average of 3 determinations after 72 h exposure, with 30 nymphs and 20 adults per replication).

Compound	Stage	LC_50_ (95% CI) (mg/L)	LC_90_ (95% CI) (mg/L)	Slope (±SE)	χ^2^ (df, *P*)
Carvacrol	Nymph	56.74 (50.19–64.53)	95.46 (83.41–110.63)	3.64 ± 0.21	6.136 (5, 0.293)
	Adults	75.62 (68.17–83.80)	148.87 (128.41–177.81)	4.45 ± 0.63	3.755 (5, 0.585)
Citral	Nymph	89.12 (76.56–104.10)	156.33 (138.85–184.25)	2.19 ± 0.43	2.209 (5, 0.697)
	Adults	102.74 (89.10–119.23)	171.70 (151.79–202.33)	3.50 ± 0.61	1.606 (5, 0.808)
Thymol	Nymph	28.52 (23.99–32.51)	71.46 (62.31–86.65)	2.87 ± 0.41	3.516 (6, 0.475)
	Adults	42.12 (34.44–49.07)	85.57 (75.67–100.61)	2.93 ± 0.43	6.559 (5, 0.161)
2-Isopropylphenol	Nymph	71.41 (60.71–83.53)	143.72 (124.51–179.73)	4.47 ± 0.65	2.937 (4, 0.568)
	Adults	85.77 (74.89–95.79)	162.53 (139.07–189.23)	4.36 ± 0.63	2.455 (4, 0.653)
3-Isopropylphenol	Nymph	82.49 (71.65–92.42)	168.18 (143.45–188.39)	4.13 ± 0.61	2.999 (5, 0.558)
	Adults	104.65 (95.93–114.08)	182.90 (161.48–212.23)	4.59 ± 0.67	4.024 (5, 0.546)
4-Isopropylphenol	Nymph	111.28 (97.51–125.46)	192.23 (173.73–225.38)	5.61 ± 0.89	5.369 (5, 0.373)
	Adults	122.36 (97.36–144.81)	211.94 (192.19–246.39)	4.87 ± 0.72	5.426 (5, 0.366)
Negative control	Nymph	—	—	—	—
	Adults	—	—	—	—

## References

[b1] Xu C. Q., Liang A. P. & Jiang G. M. The genus *Euricanla melichar* (Hemiptera: Ricaniidae) from china. Raffles Bull. Zool. 54, 1–10 (2006).

[b2] Rahman M. A. . The genus *Pochazia* amyot and serville (Hemiptera:Ricaniidae) from Korea, with a newly recorded species. J. Entomol. 9, 239–247 (2012).

[b3] Bakkali F., Averbeck S., Averbeck D. & Idaomar M. Biological effects of essential oils-a review. Food Chem. Toxicol. 46, 446–475 (2008). 17996351 10.1016/j.fct.2007.09.106

[b4] Ryu T. H. Essential oils with repellent effect against *Pochazia shatungensis* (Hemiptera: Ricaniidae). (Master’s thesis, Chungnam National University, Korea, 2014).

[b5] Senthil-Nathan S. Physiological and biochemical effect of neem and other Meliaceae plants secondary metabolites against Lepidopteran insects. Front Physiol 4, 1–17 (2013). 24391591 10.3389/fphys.2013.00359 PMC3868951

[b6] Pavela R. Essential oils for the development of eco-friendly mosquito larvicides: a review. Ind Crops Prod. 76, 174–187 (2015).

[b7] Kim M. G., Yang J. Y. & Lee H. S. Acaricidal potentials of active properties isolated from *Cynanchum paniculatum* and acaricidal changes by introducing functional radicals. J. Agric. Food Chem. 61, 7568–7573 (2013). 23855621 10.1021/jf402330p

[b8] Lee H. W. & Lee H. S. Acaricidal potency of active constituent isolated from *Mentha piperita* and its structural analogs against pyroglyphid mites. J. Korean Soc. Appl. Biol. Chem. 58, 597–602 (2015).

[b9] Yang J. Y., Kim M. G., Lee S. E. & Lee H. S. Acaricidal activities against house dust mites of spearmint oil and its constituents. Planta Med. 80, 165–170 (2014). 24488719 10.1055/s-0033-1360313

[b10] Senthil-Nathan S. A review of biopesticides and their mode of action against insect pests. In Environmental Sustainability (pp. 49–63). Springer: India (2015).

[b11] Akhtar Y., Isman M. B., Lee C. H., Lee S. G. & Lee H. S. Toxicity of quinones against two-spotted spider mite and three species of aphids in laboratory and greenhouse conditions. Ind. Crop Prod. 37, 536–541 (2012).

[b12] Venskutonis P. R. Effect of drying on the volatile constituents of thyme (*Thymus vulgaris* L.) and sage (*Salvia officinalis* L.). Food Chem. 59, 219–227 (1997).

[b13] Hendriks M. W. B., Juarez L. C., Bont D. D. & Hall R. D. Preprocessing and exploratory analysis of chromatographic profiles of plant extracts. Anal. Chim. Acta. 545, 53–64 (2005).

[b14] Lima R. K. . Chemical composition and fumigant effect of essential oil of *Lippia sidoides* Cham. and monoterpenes against Tenebrio molitor (L.) (Coleoptera: Tenebrionidae). Cienc. Agrotec. 35, 664–671 (2011).

[b15] Medeiros M. G. F. . *In vitro* antileishmanial activity and cytotoxicity of essential oil from *Lippia sidoides* Cham. Parasitol. Int. 60, 237–241 (2011). 21421075 10.1016/j.parint.2011.03.004

[b16] Bekele J. & Hassanali A. Blend effects in the toxicity of the essential oil constituents of *Ocimum Kilimandscharicum* and *Ocimum kenyenst* (Labiateae) on two post-harvest insect pests. Phytochemistry. 57, 385–391 (2001). 11393518 10.1016/s0031-9422(01)00067-x

[b17] Tiwary M., Naik S. N., Tewary D. K., Mittal P. K. & Yadav S. Chemical composition and lavicidal activities of the essential oil of *Zanthoxylum armatum* DC (Rutaceae) against three mosquito vectors. J. Vect Borne Dis. 44, 198–204 (2007). 17896622

[b18] Omolo M. O., Okinyo D., Ndiege I. O., Lwande W. & Hassanali A. Fumigant toxicity of the essential oils of some African plants against *Anopheles gambiae* sensu stricto. Phytomedicine. 12, 241–246 (2005). 15830848 10.1016/j.phymed.2003.10.004

[b19] Yousep H. & El-lakwah S. F. Effects of *Melia azedarach* ripe fruit extract on some enzyme activities of the cotton leafworm *Spodoptera littoralis* (Boised.). Egypt. J. Biol. Pest Control. 24, 315–320 (2014).

[b20] Sethil-Nathan S. Effects of *Melia azedarach* on nutritional physiology and enzyme activities of the rice leaffolder *Cnaphalocrocis medinalis* (Guenée) (Lepidoptera: Pyralidae). Pestic Biochem Physde. 84, 98–108 (2006).

[b21] Jeon J. H., Lee S. G. & Lee H. S. Isolation of insecticidal constituent from *Ruta graveolens* and structure-activity relationship studies against stored-food pests (Coleoptera). J. Food Protect. 78, 1536–1540 (2015). 10.4315/0362-028X.JFP-15-111 26219367

[b22] Walter M., Jaspers M. V., Eade K., Frampton C. M. & Stewart A. Control of *Botrytis cinerea* in grape using thyme oil. Australas Plant Pathol. 30, 21–25 (2001).

[b23] Sigma-Aldrich. In Material Safety Data Sheet (MSDS): Toxicological Information, Section 11 (Sigma-Aldrich, St. Louis, USA, 2010).

